# Loss of GPR40 in LDL receptor-deficient mice exacerbates high-fat diet-induced hyperlipidemia and nonalcoholic steatohepatitis

**DOI:** 10.1371/journal.pone.0277251

**Published:** 2022-11-04

**Authors:** Zhongyang Lu, Yanchun Li, Ai-Jun Li, Wing-Kin Syn, Stephen A. Wank, Maria F. Lopes-Virella, Yan Huang

**Affiliations:** 1 Division of Endocrinology, Diabetes and Metabolic Diseases, Department of Medicine, Medical University of South Carolina, Charleston, South Carolina, United States of America; 2 Programs in Neuroscience, Washington State University, Pullman, Washington, United States of America; 3 Division of Gastroenterology and Hepatology, Medical University of South Carolina, Charleston, South Carolina, United States of America; 4 Ralph H. Johnson Veterans Affairs Medical Center, Charleston, South Carolina, United States of America; 5 Department of Physiology, Faculty of Medicine and Nursing, University of the Basque Country, Euskal Herriko Unibertsitatea/Universidad del País Vasco, Leioa, Spain; 6 National Institute of Diabetes and Digestive and Kidney Diseases, Bethesda, Maryland, United States of America; Tokyo University of Agriculture, JAPAN

## Abstract

GPR40, a G protein-coupled receptor for free fatty acids (FFAs), is considered as a therapeutic target for type 2 diabetes mellitus (T2DM) since GPR40 activation in pancreatic beta cells enhances glucose-stimulated insulin secretion. Nonalcoholic fatty liver disease (NAFLD) is a common complication of T2DM or metabolic syndrome (MetS). However, the role of GPR40 in NAFLD associated with T2DM or MetS has not been well established. Given that it is known that cholesterol and FFAs are critically involved in the pathogenesis of nonalcoholic steatohepatitis (NASH) and LDL receptor (LDLR)-deficient mice are a good animal model for human hyperlipidemia including high cholesterol and FFAs, we generated GPR40 and LDLR double knockout (KO) mice in this study to determine the effect of GPR40 KO on hyperlipidemia-promoted NASH. We showed that GPR40 KO increased plasma levels of cholesterol and FFAs in high-fat diet (HFD)-fed LDLR-deficient mice. We also showed that GPR40 KO exacerbated HFD-induced hepatic steatosis, inflammation and fibrosis. Further study demonstrated that GPR40 KO led to upregulation of hepatic CD36 and genes involved in lipogenesis, fatty acid oxidation, fibrosis and inflammation. Finally, our *in vitro* mechanistic studies showed that while CD36 was involved in upregulation of proinflammatory molecules in macrophages by palmitic acid (PA) and lipopolysaccharide (LPS), GPR40 activation in macrophages exerts anti-inflammatory effects. Taken together, this study demonstrated for the first time that loss of GPR40 in LDLR-deficient mice exacerbated HFD-induced hyperlipidemia, hepatic steatosis, inflammation and fibrosis potentially through a CD36-dependent mechanism, suggesting that GPR40 may play a beneficial role in hyperlipidemia-associated NASH in LDLR-deficient mice.

## Introduction

GPR40, also known as free fatty acid (FFA) receptor-1, is a G protein-coupled receptor for medium- and long-chain fatty acids [1, 2]. GPR40 is highly expressed in pancreatic β-cells and mediates FFA-augmented glucose-stimulated insulin secretion (GSIS) [3]. In addition, GPR40 is expressed in enteroendocrine cells and mediates FFA-stimulated secretion of incretin that also increases insulin production from pancreatic β-cells [4]. Since type 2 diabetes mellitus (T2DM) is associated with not only insulin resistance but also β-cell dysfunction and impaired insulin secretion [5], GPR40 has been considered as a therapeutic target for T2DM by increasing insulin secretion [6, 7].

In addition to pancreatic β-cells and enteroendocrine cells, GPR40 is also expressed by many different types of cells including neuron [8], microglia [9], osteoblast [10], fibroblast [11], macrophage [11] and hepatocyte [12, 13]. GPR40 has attracted an increasing research interest since studies have shown that GPR40 is also involved in metabolic syndrome (MetS), cardiovascular diseases and nonalcoholic fatty liver disease (NAFLD) by mediating FFA uptake and FFA-activated G protein-mediated signal transduction [14, 15].

NAFLD is the most common chronic liver disorder [16–19] and a complication of T2DM or MetS [20–23]. NAFLD ranges from triglyceride accumulation (steatosis) to nonalcoholic steatohepatitis (NASH), which is characterized by steatosis, hepatic inflammation and hepatocellular ballooning, and may progress to liver fibrosis, cirrhosis, and liver cancer [24]. Since GPR40 is considered as a therapeutic target for T2DM and NAFLD is a T2DM complication, it is important to determine the role of GPR40 in pathogenesis of NAFLD. However, although a number of the previous studies have investigated the role of GPR40 in hepatic steatosis and inflammation [25–27], the findings are controversial and the role of GPR40 in NAFLD associated with T2DM or MetS has not been well established.

In the investigation of the mechanisms involved in the progression of NAFLD, it was found that free cholesterol is a major lipotoxic molecule critical for the development of experimental and human NASH [28, 29]. Musso et al. reported that cholesterol accumulation injured hepatocytes by disrupting mitochondrial and endoplasmic reticulum (ER) membrane integrity and triggering ER stress [30]. In addition, hepatic accumulation of oxidized LDL, which contains a large amount of cholesterol and is a ligand for CD36, activates Kupffer and hepatic stellate cells, and promotes liver inflammation and fibrogenesis [30]. Besides cholesterol, FFA in particular saturated fatty acid (SFA) also plays a crucial role in the pathogenesis of NASH. It has been shown that SFA induces lipotoxicity by increasing reactive oxygen species, which is associated with damaged mitochondria and hepatic inflammation and injury [31, 32]. Although it has been well documented that high cholesterol and FFAs contribute to the pathogenesis of NASH [33, 34], the role GPR40 in the progression of NAFLD promoted by high cholesterol and FFAs remains unknown.

It has been reported that LDL receptor (LDLR) knockout (KO) mice, an animal model for human HFD-induced hyperlipidemia including high cholesterol and FFAs [35], are a good animal model for studies on hyperlipidemia-related NASH [36]. In the current study, we generated GPR40 and LDLR double KO (DKO) mice and studied the role of GPR40 KO in high-fat diet (HFD)-induced NASH in hyperlipidemic LDLR-deficient mice. Interestingly, results showed that GPR40 KO in LDLR-deficient mice worsened HFD-promoted hyperlipidemia and hepatic steatosis, inflammation and fibrosis.

## Materials and methods

### Animals and diets

The animal protocol for this study was approved by the Institutional Animal Care and Use Committee (IACUC) at VA Medical Center in Charleston, SC. The GPR40-targeted KO mice were developed by replacing the GPR40 coding region with a 21-nucleotide DNA fragment encoding genes for 9 amino acids of influenza hemagglutinin antigen eGFP and neomycin as described previously [37]. LDLR-deficient mice on a C57BL/6 background were purchased from Jackson Laboratory (Bar Harbor, ME). GPR40 KO mice were crossed with LDLR KO mice to generate GPR40/LDLR DKO mice and LDLR KO littermates. All mice were maintained on a 12-hour light-dark cycle in a pathogen-free environment and had ad libitum access to water and food. The mice were randomly divided into 4 groups (n = 6 in Group 1; n = 8 in Groups 2–4, total 28 mice): 1. LDLR KO mice fed LFD (control); 2. DKO mice fed LFD; 3. LDLR KO mice fed HFD; 4. DKO mice fed HFD. The sample size was determined using power analysis based on the data from our previous study [13]. For randomization, we first randomly allocated 2 groups of LDLR KO mice or GPR40/LDLR DKO mice and then randomly allocated 2 groups of mice to LFD-fed group (LFD-fed alone) and HFD-fed group. GPR40/LDLR DKO mice or LDLR KO mice were fed either low-fat diet (LFD) (D12450B) or HFD (D12492) (Research Diet Inc., New Brunswick, NJ) for 20 weeks. D12492 is a lard based HFD containing 60% of kcal fat, 254.5 g/kg fatty acid, 81.5 g/kg saturated fatty acid (SFA) and 51 g/kg palmitic acid (PA); D12450B is contains 10% kcal fat, 43.7 g/kg fatty acid, 9.9 g/kg SFA and 1.1 g/kg PA. After the feeding, blood metabolic parameters and livers in 4 groups (6–8 mice per group) were analyzed. The animal study was conducted in Animal Facility at VA Medical Center in Charleston, SC and the methods of euthanasia to be used was consistent with the American Veterinary Medical Association (AVMA) Guideline for the Euthanasia of Animals.

### Metabolic assays

Blood samples were obtained under the fasted condition and glucose level was determined using a Precision QID glucometer (MediSense Inc., Bedford, MA). Serum cholesterol and triglycerides were assayed using Cholestech LDX Lipid monitoring System (Fisher Scientific, Pittsburgh PA). Serum fatty acids were determined using the EnzyChrom^™^ FFA kit (BioAssay systems, Hayward, CA). Serum fasting insulin was assayed using the Ultra Sensitive Insulin ELISA Kit (Crystal Chem, Inc., Downers Grove, IL, USA). Fasting whole-body insulin sensitivity was estimated with the homeostasis model assessment of insulin resistance (HOMA-IR) according to the formula [fasting plasma glucose (mg/dL) x fasting plasma insulin (μU/mL)]/405. Serum alanine transaminase (ALT) was assayed using an ALT assay kit (Cayman Chemical, Ann Arbor MI).

### Histological examination of liver tissue

The liver tissue was embedded in Tissue-Tek^®^ OCT^™^ compound (EMS, Hatfield, PA), immediately frozen on dry ice, and stored at -80°C. The tissue with 6 μm thickness was sectioned and mounted on glass microscope slides. The sections were fixed in 10% of formalin and stained with Harris modified hematoxylin and eosin (H&E) solution (Sigma, St. Louis, MO). Slides were dehydrated and mounted in Cytoseal-XYL mounting medium (Fisher Scientific, Waltham, MA). Photomicrographs of tissue sections were taken using an Olympus BX53 digital microscope with Cellsens digital image software (Olympus American Inc., Center Valley, PA).

### Oil Red O and Sirius Red staining

For Oil Red O staining, the frozen sections were fixed with 10% formalin for 10 min, placed in 60% isopropyl alcohol and stained in 0.5% Oil Red O solution for 10 min. The slides were transferred to 60% isopropyl alcohol, rinsed in distilled water and processed for hematoxylin counter staining. For Sirius Red staining, the sections were fixed with 10% formalin for 10 min, incubated with a 0.1% Sirius Red solution dissolved in aqueous saturated picric acid for 1 h, washed in acidified water (0.5% acetic acid), dehydrated, and mounted on slides. Photomicrographs of tissue sections were taken using an Olympus BX53 digital microscope and the positively stained area was quantified with ImageJ (NIH, Bethesda, MD) and presented as percent of total area of the examined field.

### Immunohistochemical analysis of F4/80 expression

Liver tissues were fixed in 4% paraformaldehyde for 10 min and frozen sections were made using a cryostate. Immunohistochemical analysis with anti-F4/80 antibodies (Category number: MCA497, Bio-Rad Laboratories, Inc., Hercules, CA) was performed as described previously [38]. Counterstaining was performed with hematoxylin. Photomicrographs of tissue sections were taken using an Olympus BX53 digital microscope and the positively immunostained area was quantified with ImageJ (NIH, Bethesda, MD) and presented as percent of total area of the examined field.

### Cell culture and treatment

Bone marrow–derived macrophages were prepared and cultured as described previously [39]. Briefly, bone marrow cells were obtained from tibiae and femora of 4- to 6-wk-old C57BL/6 mice and cultured with α-MEM containing 10% fetal bovine serum in a humidified incubator (5% CO2) at 37°C. After 24 h, nonadherent cells were incubated for 4 d in the presence of 50 ng/mL of macrophage colony stimulating factor (Sigma-Aldrich, Atlanta, GA, USA). It has been shown that almost all the adherent cells expressed macrophage-specific antigens, such as Mac-1, Moma-2, and F4/80 [40].

RAW 264.7 cells were purchased from American Type Culture Collection (Manassas, VA) and grown in DMEM (American Type Culture Collection, Manassas, VA) supplemented with 10% heat-inactivated fetal calf serum (HyClone, Logan, UT). The cells were maintained in a 37°C, 90% relative humidity, 5% CO2 environment. To prepare palmitic acid (PA), PA was dissolved in 0.1 N NaOH and 70% ethanol at 70°C to make PA solution at concentration of 50 mM. The solution was kept at 55°C for 10 min, mixed, and brought to room temperature. Lipopolysaccharide (LPS) from Escherichia coli (serotype 055:B5) was purchased from Sigma (St. Louis, MO). RAW 264.7 cells were treated with PA, LPS or both for 24 h. After the treatment, culture medium was collected for quantification of IL-6 protein and cells were subjected to RNA isolation for IL-6 mRNA quantification by real-time PCR. RAW264.7 macrophages were also treated with CD36 inhibitor sulfosuccinimidyl oleate (SSO), GPR40 antagonist GW1100 or GPR40 agonist GW9508 (Cayman Chemical, Ann Arbor, MI).

### Enzyme-linked immunosorbent assay (ELISA)

IL-6 in medium were quantified using sandwich ELISA kits according to the protocol provided by the manufacturer (Biolegend, San Diego, CA).

### RNA isolation from liver tissues

Total RNA was isolated from mouse liver tissues using the RNeasy minikit (Qiagen, Santa Clarita, CA) by following the instructions provided by the company.

### Real-time polymerase chain reaction (PCR)

Real-time PCR was performed as described previously [41]. The mouse CD36, GPR40, Sterol regulatory element-binding proteins (SREBP)-1c, Fatty acid synthase (FAS), carnitine palmitoyltransferase 1A (CPT1a), acyl-CoA oxidase (ACOX)1, alpha smooth muscle actin (αSMA), transforming growth factor beta 1 (TGFβ1), IL-6, tumor necrosis factor alpha (TNFα) and glyceraldehyde-3-phosphate dehydrogenase) (GAPDH) primers (Table 1) were synthesized by Integrated DNA Technologies, Inc. (Coralville, IA, USA). The PCR data were analyzed with the iCycler iQ^™^ software. The average starting quantity (SQ) of fluorescence units was used for analysis. Quantification was calculated using the SQ of targeted cDNA relative to that of GAPDH cDNA in the same sample.

**Table 1:** Sequences of primers used for real-time PCR.

Genes	Forward primer sequences	Reverse primer sequences
CD36	TGCTGGAGCTGTTATTGGTG	CATGAGAATGCCTCCAAACA
GPR40	TGGAGTCACAGAAGGAGTGGCTAAG	TCTGACCACAGTGAGGAATGTCCAC
SREBP-1c	GGAGCCATGGATTGCACATT	GGCCCGGGAAGTCACTGT
FAS	CTTCCGAGATTCCATCCTACGC	TGGCAGTCAGGCTCACAAACG
CPT1a	TCATCAGCAACCGGCCCAAA	GGAGGTTGTCCACGAGCCAG
ACOX1	CAAGACCCAAGAGTTCATT	TTCAGGTAGCCATTATCCA
aSMA	GAGGCACCACTGAACCCTAA	CATCTCCAGAGTCCAGCACA
TGFβ1	TGGTGGACCGCAACAACGCC	GGCCATGAGGAGCAGGAAGG
IL-6	TGGAGTCACAGAAGGAGTGGCTAAG	TCTGACCACAGTGAGGAATGTCCAC
TNFα	TTCCAGAACTCCAGGCGGT	TGGGCTACAGGCTTGTCACTC
GAPDH	CTGAGTACGTCGTGGAGTC	AAATGAGCCCCAGCCTTC

SREBP-1c: sterol regulatory element-binding protein-1c; FAS: fatty acid synthase; CPT1a: carnitine palmitoyltransferase 1A; ACOX1: acyl-CoA oxidase 1; αSMA; alpha smooth muscle actin; TGFβ1: transforming growth factor beta 1; TNFα: tumor necrosis factor alpha; GAPDH: glyceraldehyde-3-phosphate dehydrogenase.

### Statistical analysis

All statistical analyses were performed using GraphPad Prism 8 (v. 8.4.0) (GraphPad Software, Inc., La Jolla, CA). To determine the statistical significance of differences between two experimental groups, parametric analysis using Student’s t test was performed for data with a normal distribution, whereas nonparametric analysis using the Mann–Whitney test was performed for data without a normal distribution. For in vitro studies, the experiments were performed in duplicates for 3 times and the data were presented as Least Squares means SD. One-way ANOVA was performed to determine the statistical significance among different experimental groups. A value of P < 0.05 was considered significant.

## Results

### GPR40 KO in LDLR-deficient mice worsens HFD-induced hyperlipidemia and hepatic injury

**Fig 1:**
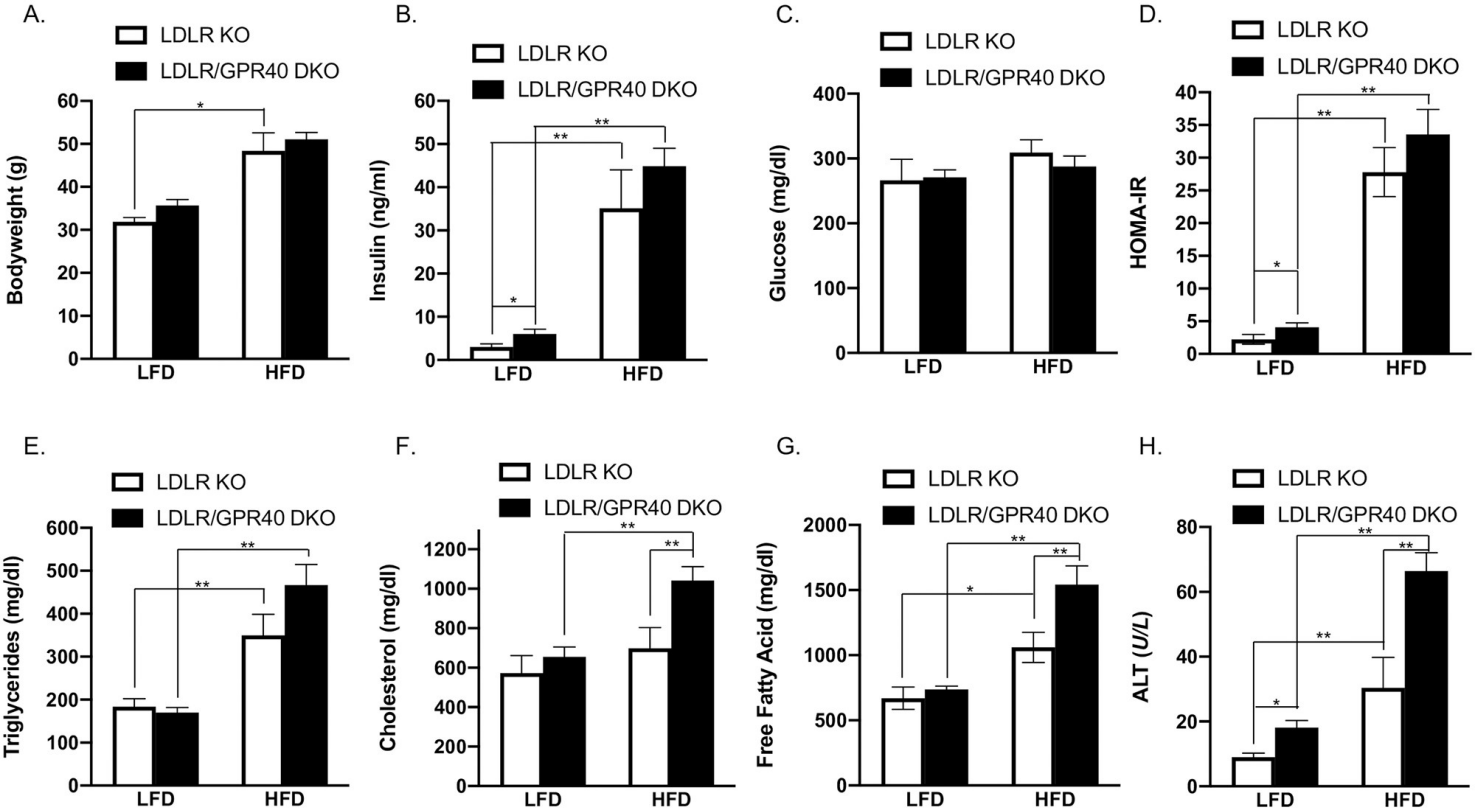
The effects of GPR40 KO on the metabolic parameters and liver injury marker in LFD- or HFD-fed LDLR-deficient mice. Eight-week-old male LDLR-deficient mice with GPR40 KO and their wild-type littermates were fed LFD or HFD for 20 wk and the following metabolic parameters were determined at the end of the experiment: (A) body weight; (B) insulin; (C) glucose;(D) insulin resistance (HOMA-IR); (E) triglycerides; (F) cholesterol; (G) free fatty acids; (H) liver injury marker ALT. The data are means±SD (n = 6–8). One-way ANOVA and Student’s t test were used for the statistical analysis. * *p*<0.05; **p<0.01. ALT, alanine aminotransferase; HOMA-IR, homeostasis model assessment of insulin resistance. HFD, high-fat diet; LFD, low-fat diet; KO, knockout; DKO, double knockout.

We first determined how GPR40 KO in LDLR-deficient mice affected metabolic parameters and hepatic injury. Results showed that HFD increased bodyweight significantly, but GPR40 KO did not further change the bodyweight (Fig 1A). While HFD markedly increased insulin (Fig 1B), it did not change glucose level (Fig 1C), suggesting the presence of insulin resistance. Consistently, HFD robustly increased HOMA-IR (Fig 1D), an index for insulin resistance. GPR40 KO slightly increased insulin and HOMA-IR in mice fed LFD, but not those fed HFD (Fig 1B and 1D) and had no effect on glucose in both LFD- and HFD-fed mice (Fig 1C). For lipids, HFD increased triglycerides (Fig 1E) and FFAs (Fig 1G), but not cholesterol (Fig 1F) while GPR40 KO augmented cholesterol and FFAs, but not triglycerides, in HFD-fed mice. In addition, while either HFD or GPR40 KO increased ALT in LFD-fed mice, GPR40 KO further increased ALT in HFD-fed mice (Fig 1H). Taken together, results from metabolic studies showed that GPR40 KO in LDLR-deficient mice augmented HFD-increased cholesterol, FFA and hepatic injury.

### GPR40 KO in LDLR-deficient mice is associated with hepatic steatosis and hepatocellular ballooning

**Fig 2:**
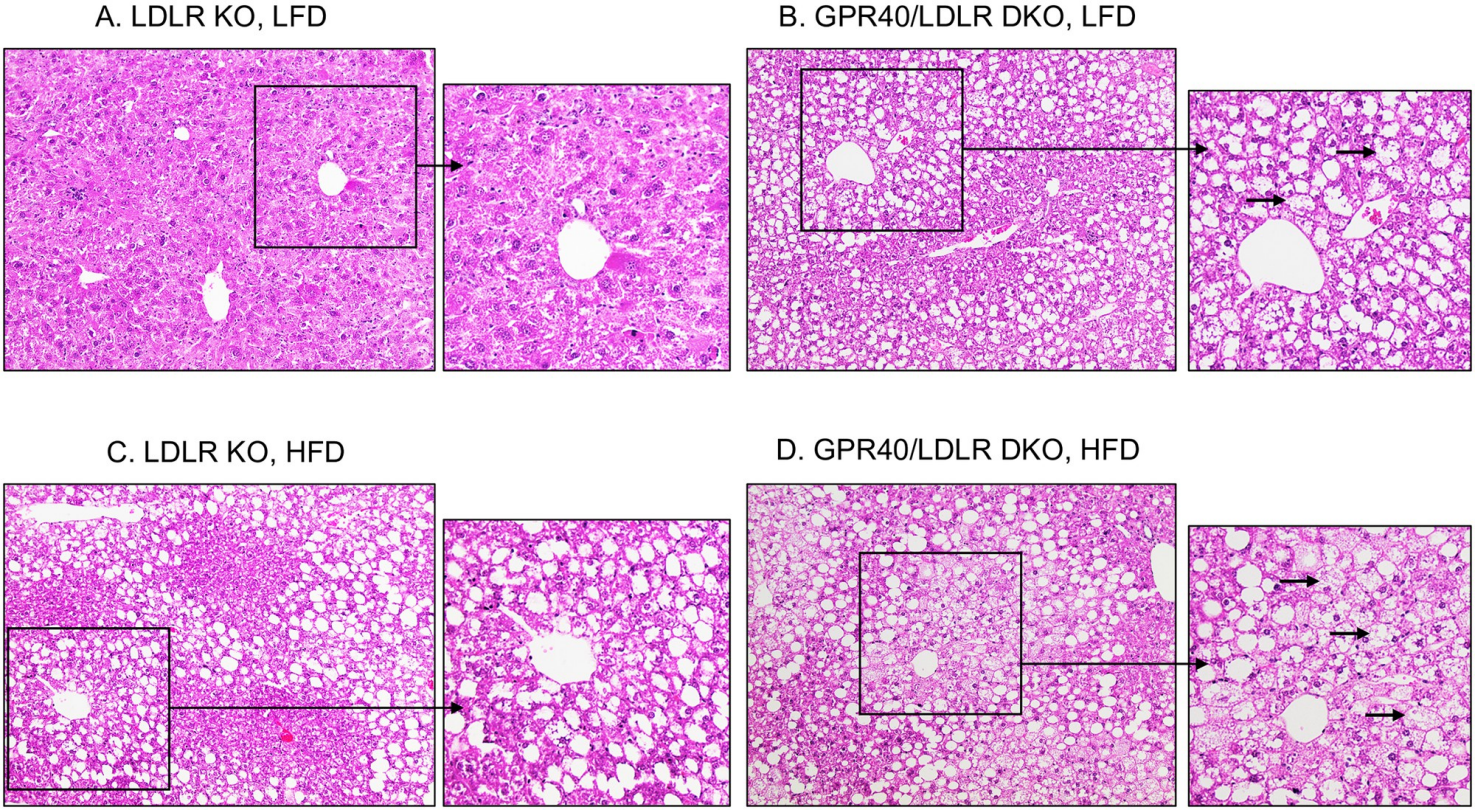
The effects of GPR40 KO on hepatic steatosis and hepatocellular ballooning in LFD- or HFD-fed LDLR-deficient mice. LDLR KO or GPR40/LDLR DKO mice were fed LFD or HFD for 20 wk. After the feeding, livers were dissected, and histological analysis was performed. A–D: Representative images of liver sections in four groups. The tissue sections were stained with hematoxylin and eosin and presented with low (x100) and high magnification (x400). The arrows indicate the enlarged hepatocytes with rarefied cytoplasm (hepatocellular ballooning). LFD, low-fat diet; HFD, high-fat diet; KO, knockout; DKO, double knockout.

**Fig 3:**
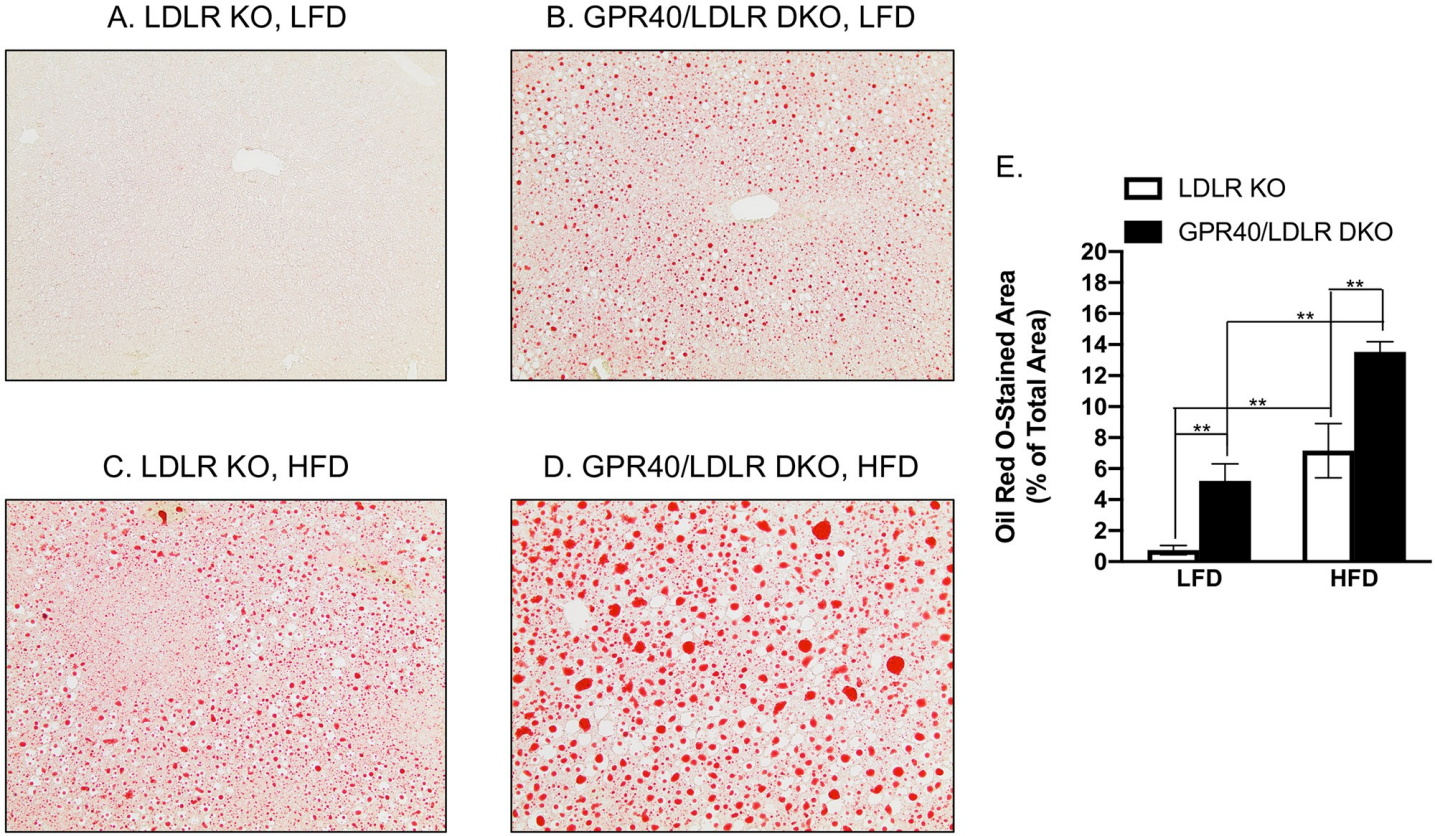
The effect of GPR40 KO on steatosis in LFD- or HFD-fed LDLR-deficient mice. A–D: Representative images of liver sections with Oil Red O staining (magnification: x100). E: The positively stained areas of Oil Red O staining in tissue sections were quantified and compared among all groups. The data are presented as means±SE (n = 6–8). One-way ANOVA and Student’s t test were used for the statistical analysis. **p<0.01. LFD, low-fat diet; HFD, high-fat diet; KO, knockout; DKO, double knockout.

We performed H&E staining of liver tissues and assessed the effect of GPR40 KO on hepatic steatosis and hepatocellular ballooning in LDLR-deficient mice. Results showed that, compared to LFD-fed LDLR KO mice (Fig 2A), LFD-fed GPR40/LDLR DKO mice is associated with steatosis and hepatocellular ballooning (Fig 2B). As expected, HFD induced hepatic steatosis in LDLR-deficient mice (Fig 2C). Interestingly, HFD-fed GPR40/LDLR DKO mice is associated with not only hepatic steatosis but also hepatocellular ballooning (Fig 2D). To quantify the amount of fat in livers, we stained fat using Oil Red O staining. Results showed that LFD-fed GPR40/LDLR DKO mice had fat accumulation (Fig 3B and 3E) compared with LFD-fed LDLR KO mice (Fig 3A and 3E). While HFD induced steatosis in LDLR KO mice (Fig 3C and 3E) as expected, HFD-fed GPR40/LDLR DKO mice had increased steatosis (Fig 3D and 3E).

### GPR40 KO is associated with hepatic inflammation

**Fig 4:**
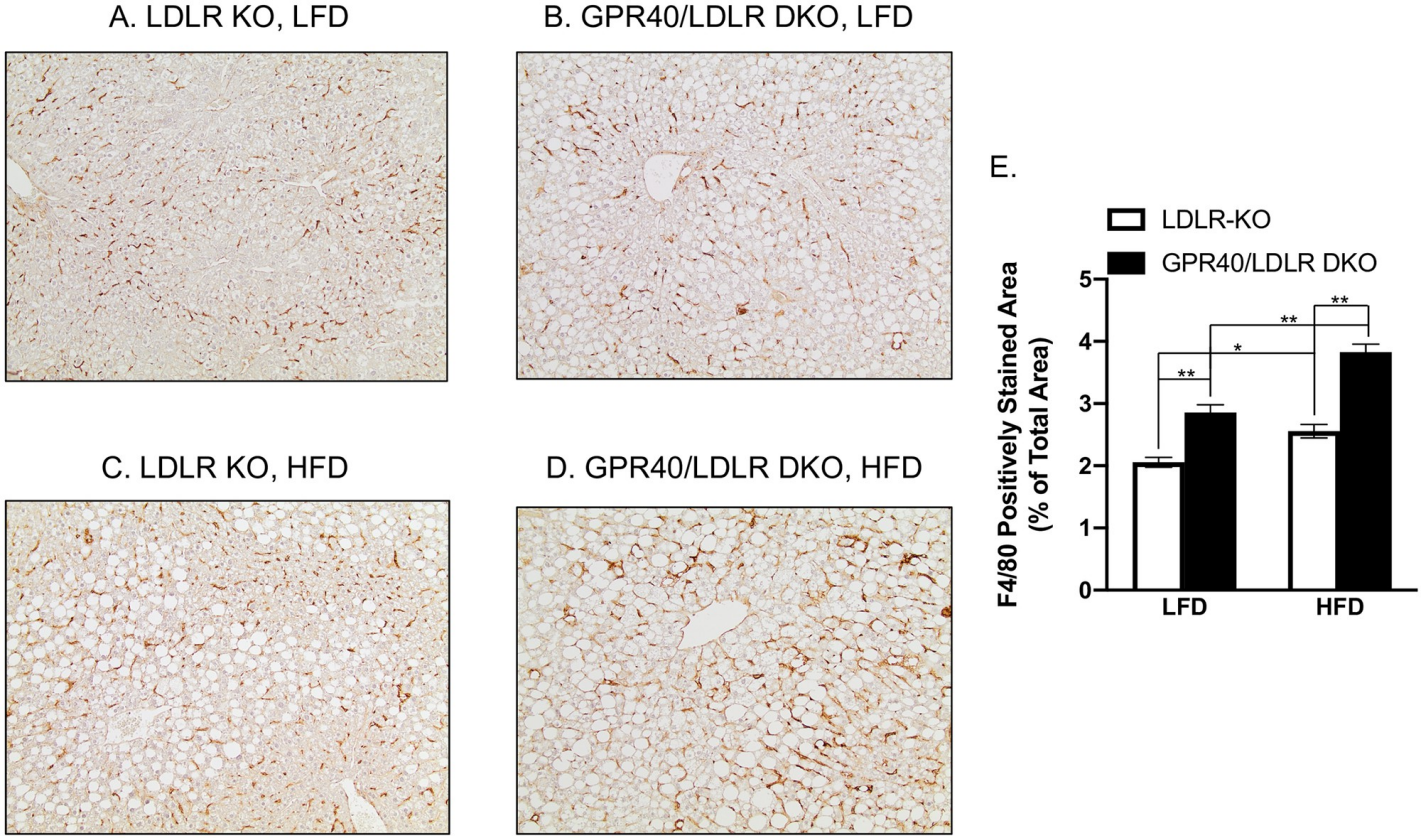
The effect of GPR40 KO on hepatic inflammation in LFD- or HFD-fed LDLR-deficient mice. A–D: Representative images of liver sections with F4/80 immunostaining (magnification: x100). E: The positively stained areas of F4/80 immunostaining were quantified and compared among all groups. The data are presented as means±SE (n = 6–8). One-way ANOVA and Student’s t test were used for the statistical analysis. *p<0.05; **p<0.01. LFD, low-fat diet; HFD, high-fat diet; KO, knockout; DKO, double knockout.

Immunohistochemical staining of F4/80, a biomarker for murine macrophages [42], was performed to assess the effect of GPR40 KO on hepatic inflammation. Results showed that LFD-fed GPR40/LDLR DKO mice had upregulated F4/80 expression (Fig 4B and 4E) compared with LFD-fed LDLR KO mice (Fig 4A). As expected, HFD induced F4/80 expression in LDLR KO mice (Fig 4C and 4E). Interestingly, HFD-fed GPR40/LDLR DKO mice had further upregulated F4/80 expression (Fig 4D and 4E).

### GPR40 KO is associated with increased hepatic fibrosis in LDLR-deficient mice

**Fig 5:**
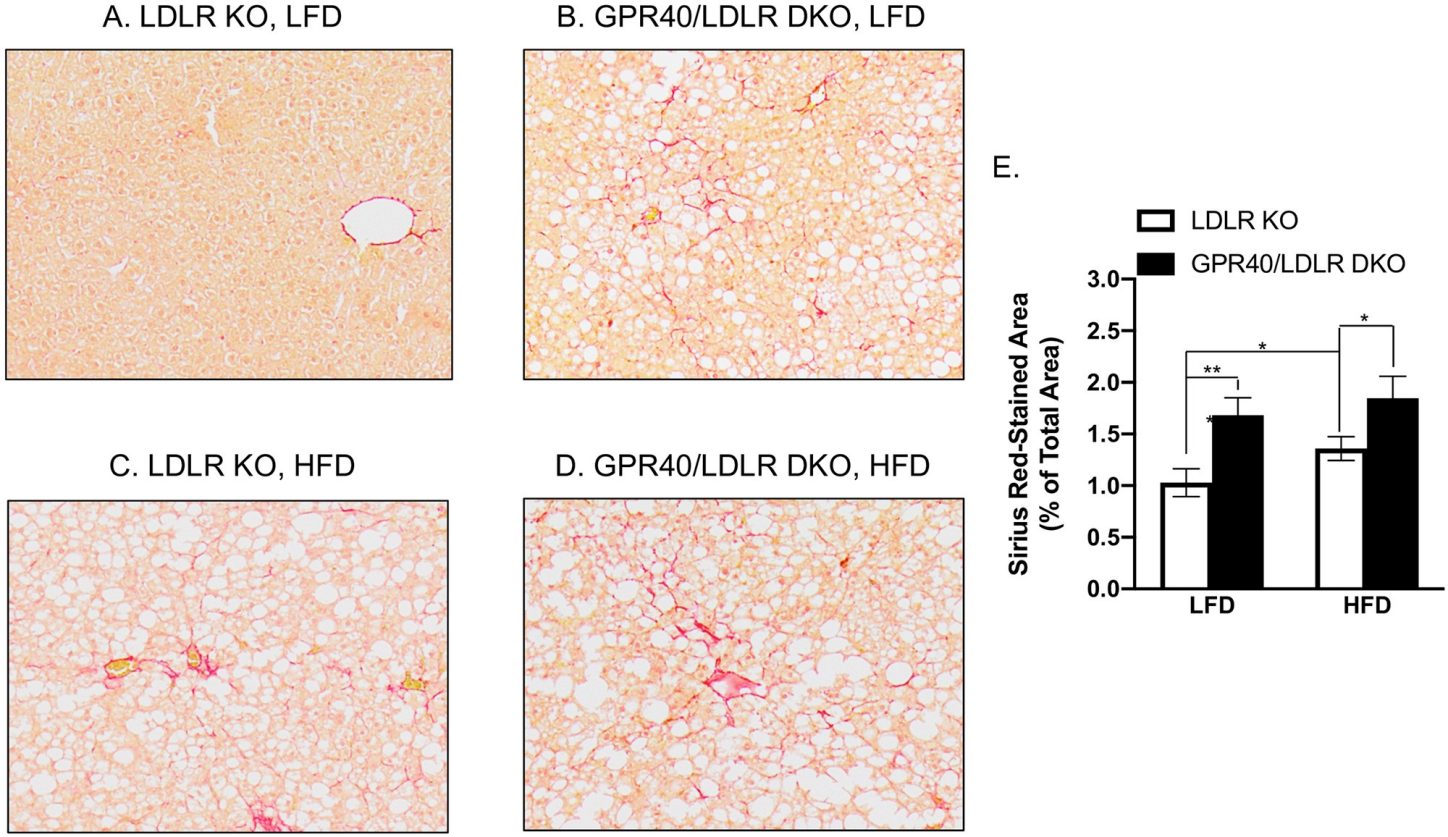
The effect of GPR40 KO on hepatic fibrosis in LFD- or HFD-fed LDLR-deficient mice. A–D: Representative images of liver sections with Sirius Red staining (magnification: x100). E: The positively stained areas of Sirius Red staining were quantified and compared among all groups. The data are presented as means±SE (n = 6–8). One-way ANOVA and Student’s t test were used for the statistical analysis. * *p*<0.05; **p<0.01. LFD, low-fat diet; HFD, high-fat diet; KO, knockout; DKO, double knockout.

Sirius Red staining was performed to assess the effect of GPR40 KO on hepatic fibrosis in LDLR KO mice. Results showed that LFD-fed GPR40/LDLR DKO mice had increased hepatic fibrosis (Fig 5B and 5E) compared with LFD-fed LDLR KO mice (Fig 5A and 5E). While HFD induced hepatic fibrosis in LDLR KO mice (Fig 5C and 5E), HFD-fed GPR40/LDLR DKO mice had further enhanced hepatic fibrosis (Fig 5D and 5E).

### GPR40 KO is associated with dysregulation of hepatic CD36 and genes involved in lipogenesis, fibrosis and inflammation

**Fig 6:**
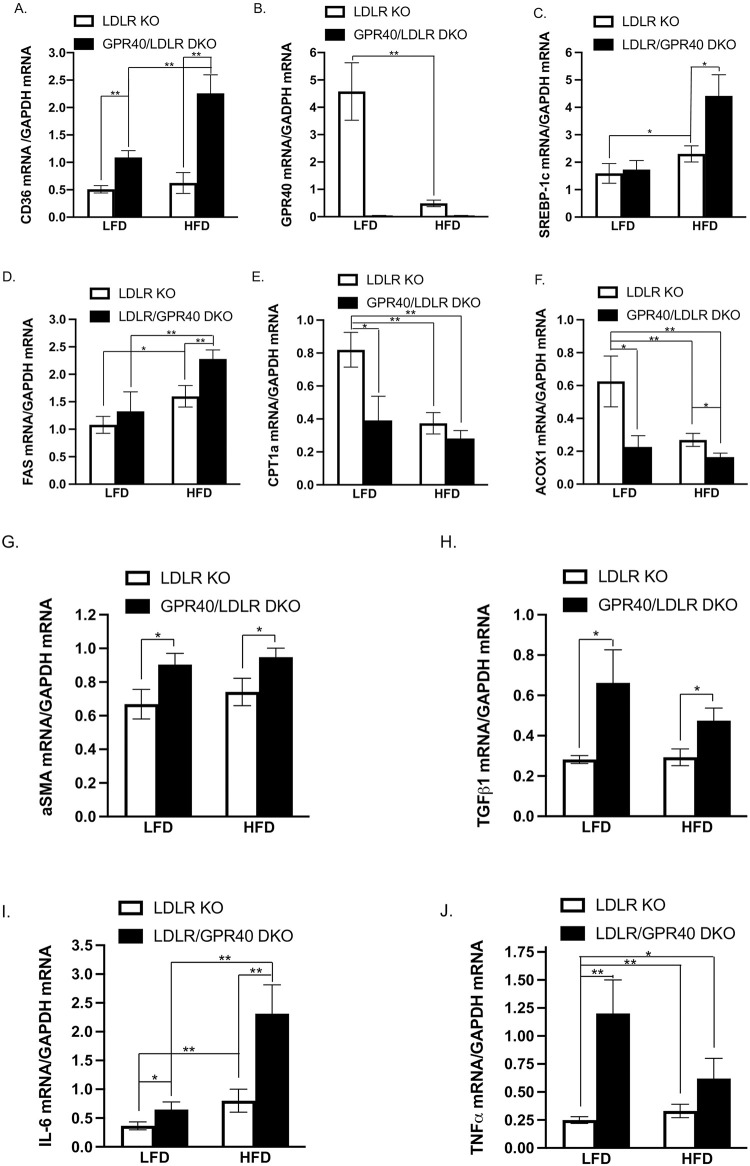
GPR40 KO is associated with upregulation of CD36 and genes involved in lipogenesis. After feeding mice an LFD or HFD for 20wk, RNA was isolated from livers of all mice and the following mRNA was quantified using real-time PCR and normalized to GAPDH mRNA: (A) CD36; (B) GPR40; (C) sterol regulatory element-binding protein (SREBP)-1c; (D) fatty acid synthase (FAS); (E) carnitine palmitoyltransferase 1A (CPT1a); (F) acyl-CoA oxidase (ACOX)1; (G) alpha smooth muscle actin (aSMA); (H) transforming growth factor beta 1 (TGFβ1); (I) IL-6; (J) tumor necrosis factor alpha (TNFα). The data are presented as means±SE (n = 6–8). One-way ANOVA and Student’s t test were used for the statistical analysis. * *p*<0.05; **p<0.01. LFD, low-fat diet; HFD, high-fat diet; KO, knockout; DKO, double knockout.

Since GPR40 is a receptor for FFAs, it is expected that GPR40 KO would lead to reduction of FFA uptake and lipid accumulation in liver. Surprisingly, the above results showed that GPR40 KO led to hepatic steatosis in LFD-fed mice and increased hepatic steatosis in HFD-fed mice. To explore the mechanisms involved in GPR40 KO-associated hepatic steatosis, we hypothesized that GPR40 KO may lead to upregulation of CD36, another receptor for FFAs [43]. To test our hypothesis, we quantified hepatic CD36 mRNA expression and results showed that GPR40 KO in LFD-fed LDLR KO mice led to a marked CD36 upregulation (Fig 6A). CD36 expression in HFD-fed LDLR KO mice appeared to be higher than that in LFD-fed LDLR KO mice, but the difference was not statistically significant (Fig 6A). Interestingly, HFD-fed GPR40/LDLR DKO mice further upregulated CD36 mRNA expression as compared to LFD-fed LDLR KO mice (Fig 6A). Results also confirmed that GPR40 expression was undetected in mice with GPR40 KO (Fig 6B). In contrast to CD36 upregulation, HFD potently downregulated GPR40 expression in LDLR KO mice, (Fig 6B).

To understand how GPR40 KO increased HFD-induced hepatic steatosis in LDLR KO mice, we quantified hepatic mRNA levels of genes involved in lipogenesis in addition to CD36. Results showed that while HFD stimulated the expression of sterol regulatory element-binding protein (SREBP)-1c and fatty acid synthase (FAS), two important molecules involved in lipogenesis (29,30), GPR40 KO further increased HFD-upregulated SREBP-1c (Fig 6C) and FAS (Fig 6D). We also quantified hepatic mRNA levels of genes involved in fatty acid oxidation since decreased expression of these genes may contribute to lipid accumulation. Results showed that either HFD or GPR40 KO reduced CPT1a and ACOX1 mRNA expression and the combination of GPR40 KO and HFD further downregulated ACOX1 mRNA expression (Fig 6E and 6F). Furthermore, we quantified mRNA of genes involved in hepatic fibrosis and inflammation such as aSMA, TGFβ1, IL-6 and TNFα. Results showed that GPR40 KO is associated with increased expression of aSMA, TGFβ1, IL-6 and TNFα in mice fed either LFD or HFD (Fig 6G–6J).

### Loss of GPR40 facilitates the stimulation by PA of IL-6 secretion from macrophages through a CD36-dependent pathway

**Fig 7:**
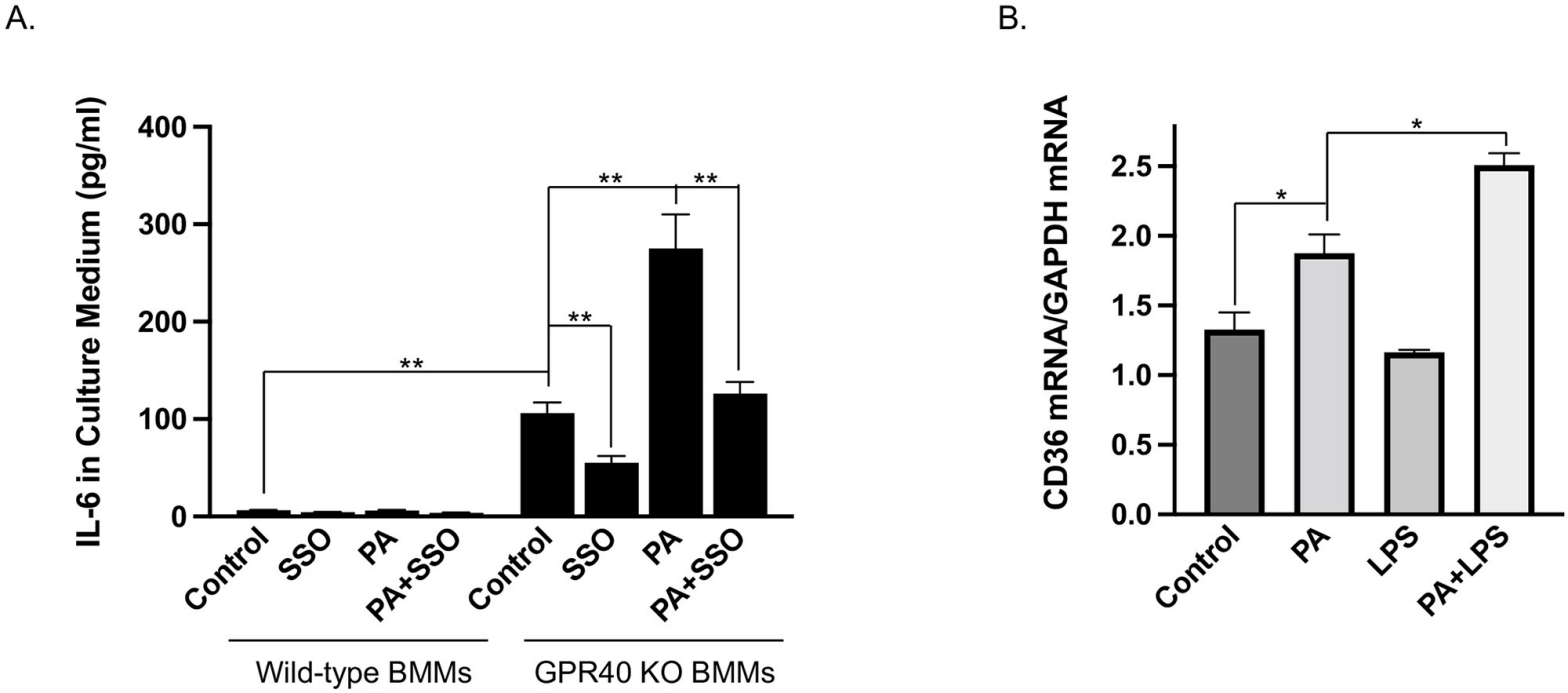
The effect of GPR40 KO on PA-stimulated IL-6 secretion by macrophages *in vitro*. A. The bone marrow cells were isolated from wild-type or GPR40 KO mice and bone marrow-derived macrophages (BMMs) were prepared and stimulated with or without 100 μM of PA in the absence or presence of 50 μM SSO for 24 h. After the treatment, IL-6 in culture medium was quantified using ELISA. B. BMMs were treated with 100 μM of PA, 0.1 ng/ml of LPS or both for 24 h and CD36 mRNA was quantified using real-time PCR as described in Methods. The presented data (means±SD) were representative of three experiments with similar results. Student’s t test was used for the statistical analysis. * *p*<0.05; **p<0.01. SSO, sulfosuccinimidyl oleate; LPS, lipopolysaccharide; PA, palmitic acid.

The above studies showed that GPR40 KO robustly increased CD36 expression in liver (Fig 6A), suggesting that the presence of GPR40 inhibits CD36 expression. To determine if CD36 upregulation resulted from GPR40 deficiency is involved in hepatic inflammation, we prepared BMMs from either wild-type or GPR40 KO mice and treated cells with PA, a common long-chain saturated fatty acid [44], in the absence or presence of CD36 inhibitor sulfosuccinimidyl oleate (SSO) [45]. Results showed that while the baseline secretion of IL-6 was very low in wild-type BMMs (6.6±0.3 ng/ml), it was increased significantly in GPR40 KO BMMs (106±11 ng/ml) (Fig 7A). Furthermore, while PA had no effect on IL-6 secretion from wild-type BMMs (6.1±0.7 ng/ml), it stimulated IL-6 secretion from GPR40 KO BMMs (275±35 ng/ml). Interestingly, CD36 inhibitor SSO inhibited PA-stimulated IL-6 secretion from GPR40 KO BMMs (126±12 ng/ml) (Fig 7A), indicating that PA-stimulated IL-6 secretion from GPR40 KO BMMs is CD36-dependent. We further determined the effect of PA on CD36 expression in BMMs. Results showed that PA significantly upregulated CD36 expression and the combination of PA and LPS further upregulated CD36 expression (Fig 7B). These findings suggest that although PA upregulates CD36 expression, the presence of GPR40 inhibits PA-elicited CD36 signaling for IL-6 expression in macrophages.

### The different effects of GPR40 and CD36 inhibition on IL-6 expression in response to PA and LPS

**Fig 8:**
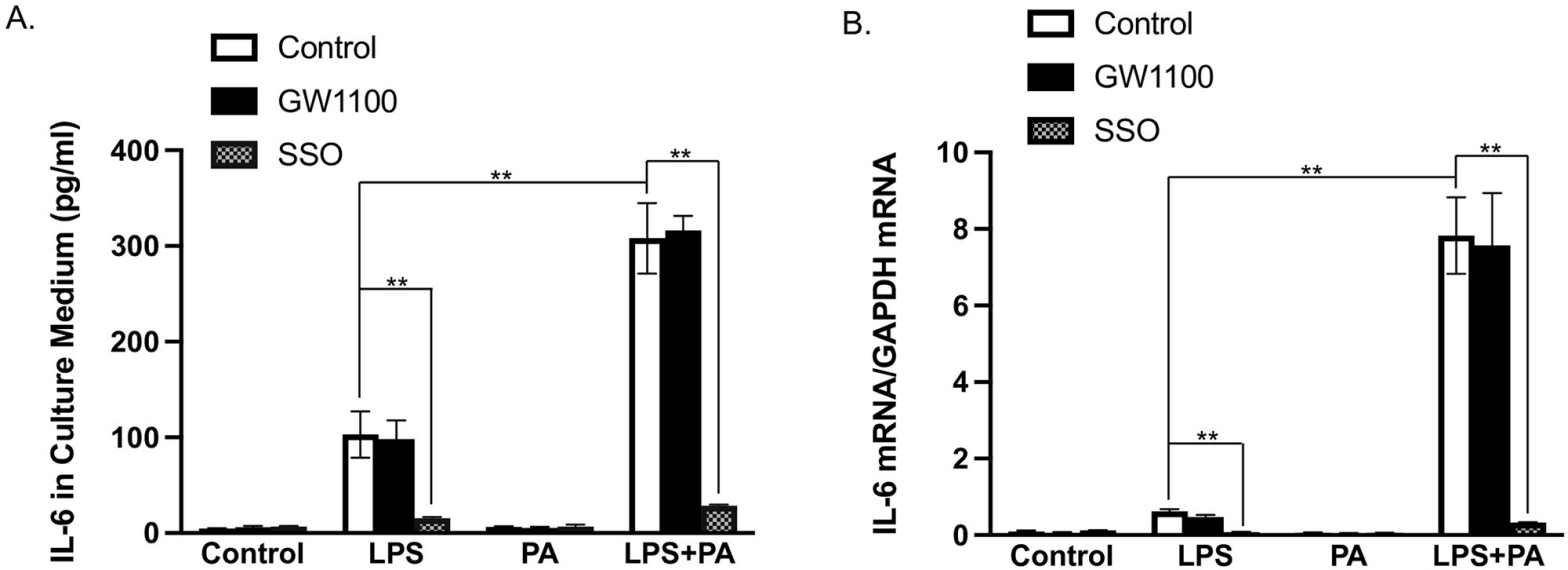
The effect of GPR40 antagonist GW1100 or CD36 inhibitor SSO on IL-6 secretion and expression in response to LPS, PA or both in macrophages. RAW264.7 macrophages were treated with 1 ng/ml LPS, 100 μM of PA or both in the absence or presence of 5 μM GW1100 or 50 μM SSO for 24 h. After the treatment, the culture medium was collected for IL-6 ELISA (A) and total RNA was isolated from cells to quantify IL-6 mRNA using real-time PCR (B). The presented data (means±SD) were representative of three experiments with similar results. Student’s t test was used for the statistical analysis. * *p*<0.05; **p<0.01. SSO, sulfosuccinimidyl oleate; LPS, lipopolysaccharide; PA, palmitic acid.

We have shown previously that PA amplifies LPS signaling in the upregulation of proinflammatory genes such as IL-6 in RAW264.7 macrophages [41]. To determine which receptor, CD36 or GPR40, mediates PA’s effect on IL-6 expression, we treated RAW264.7 macrophages with LPS, PA or LPS plus PA in the presence or absence of CD36 inhibitor SSO or GPR40 antagonist GW1100 [46]. Results showed that, although PA alone had no effect on IL-6 secretion and expression, it markedly augmented LPS-induced IL-6 secretion (Fig 8A) and mRNA expression (Fig 8B). Results further showed that SSO strongly inhibited IL-6 secretion and mRNA expression stimulated with PA and LPS, but GW1100 has no effect (Fig 8A and 8B), suggesting that CD36, but not GPR40, is involved in the amplification of LPS-induced IL-6 expression by PA.

### GPR40 activation inhibits proinflammatory cytokine secretion from macrophages

**Fig 9:**
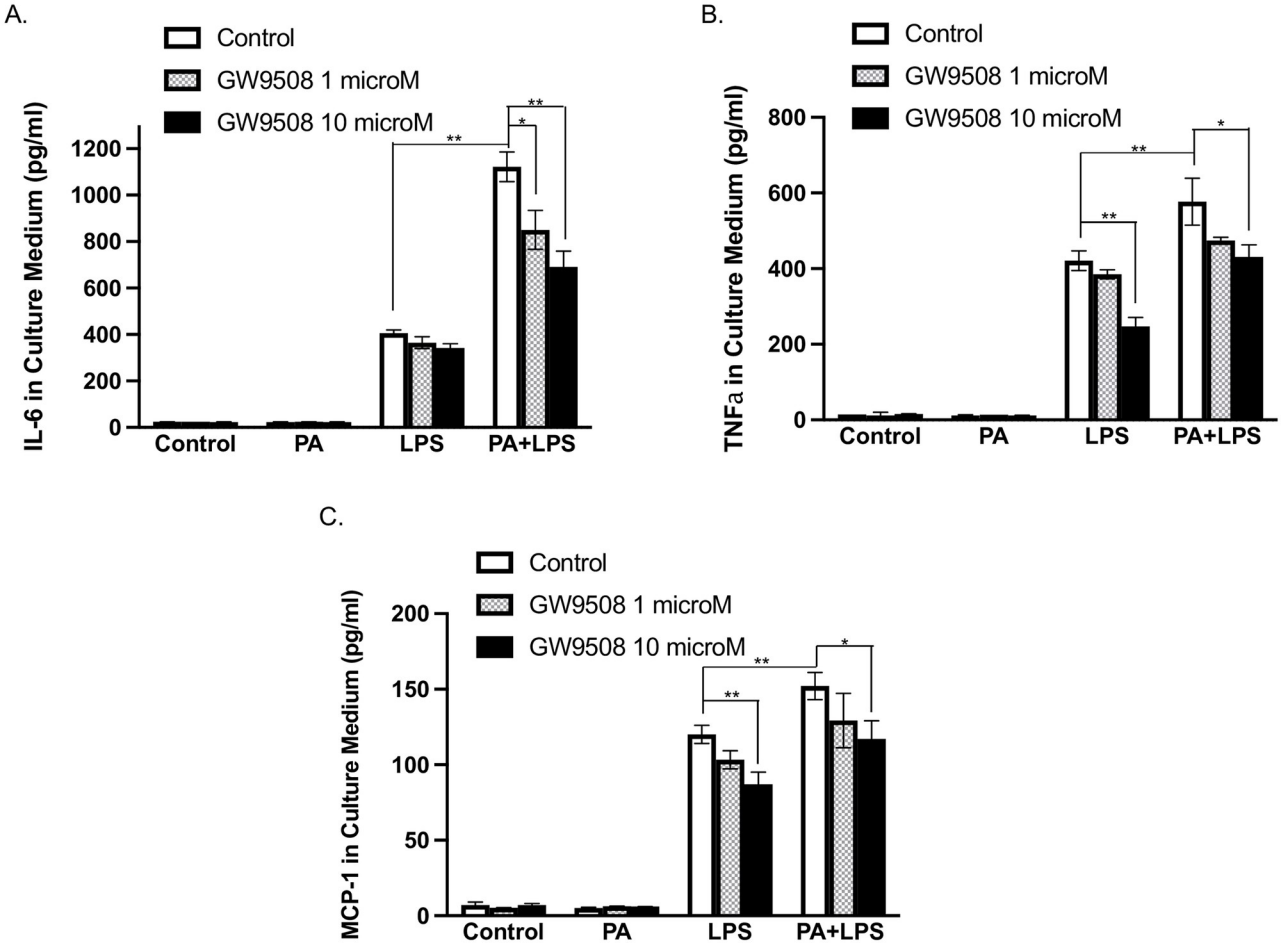
The effect of GPR40 agonist on proinflammatory cytokine expression stimulated by PA or PA plus LPS in macrophages. RAW264.7 macrophages were treated with 100 μM of PA, 1 ng/ml of LPS or both in the absence or presence of 1 or 10 μM of GW9508 for 24 h. After the treatment, the culture medium was collected for ELISA for quantifing IL-6 (A), TNFα (B) and MCP-1 (C). The presented data (means±SD) were representative of three experiments with similar results. Student’s t test was used for the statistical analysis. * *p*<0.05; **p<0.01. PA, palmitic acid; LPS, lipopolysaccharide.

To further understand the role of GPR40 on hepatic inflammation, we determined the effect of GPR40 activation on proinflammatory cytokine expression in macrophages. Results showed that GPR40 agonist GW9508 [47] dose-dependently reduced the protein secretion of IL-6, MCP-1 and TNFα in macrophages stimulated with LPS or PA plus LPS (Fig 9), supporting the hypothesis that GPR40 has anti-inflammatory property.

## Discussion

The metabolic assays in the current and our previous studies [13] showed that LDLR-deficient mice had much higher levels of plasma lipids such as cholesterol and FFA than C57BL/6 mice after HFD feeding for 20 weeks. For examples, the plasma level of cholesterol was about 700 mg/dl in LDLR-deficient mice and 250 mg/dl in C57BL/6 mice, and free fatty acid was about 1000 mg/dl in LDLR-deficient mice and 500 mg/dl in C57BL/6 mice. Since it has been well documented that high levels of cholesterol and FFAs play a key role in the hepatic inflammation and the progression of NAFLD from hepatic steatosis to NASH [33, 34, 48, 49], it is obvious that LDLR-deficient mice are a better model than C57BL/6 mice in the investigation of pathogenesis of hyperlipidemia-associated NASH. Indeed, hyperlipidemic LDLR-deficient mice have been used previously as the animal model for human NASH [48, 50–52].

The present study showed that GPR40 KO in LDLR-deficient mice exacerbated HFD-induced hepatic steatosis, inflammation and fibrosis. In contrast, our previous study showed that GPR40 KO in HFD-fed C57BL/6 mice did not further increase hepatic steatosis although it did increase hepatic inflammation [13]. To explain the different effects of GPR40 KO on hepatic steatosis in LDLR-deficient mice and C57BL/6 mice, we postulated that the elevated plasma levels of FFA and cholesterol, which were observed only in GPR40 KO in LDLR-deficient mice, are responsible for the different effects of GPR40 on hepatic steatosis.

Interestingly, while GPR40 KO only increased plasma FFA and cholesterol in LDLR-deficient mice but not C57BL/6 mice, it upregulated hepatic CD36 in both mouse models, suggesting that the presence of hepatic GPR40 inhibits CD36 expression in both LDLR-deficient mice and C57BL/6 mice. Since it has been well established that CD36 is associated with insulin resistance, hepatic steatosis and inflammation [53–56], and obesity-related lipotoxicity [57], the upregulation of CD36 as a result of GPR40 KO is likely to play a pivotal role in GPR40 KO-promoted hepatic steatosis, inflammation and fibrosis in LDLR-deficient mice.

**Fig 10:**
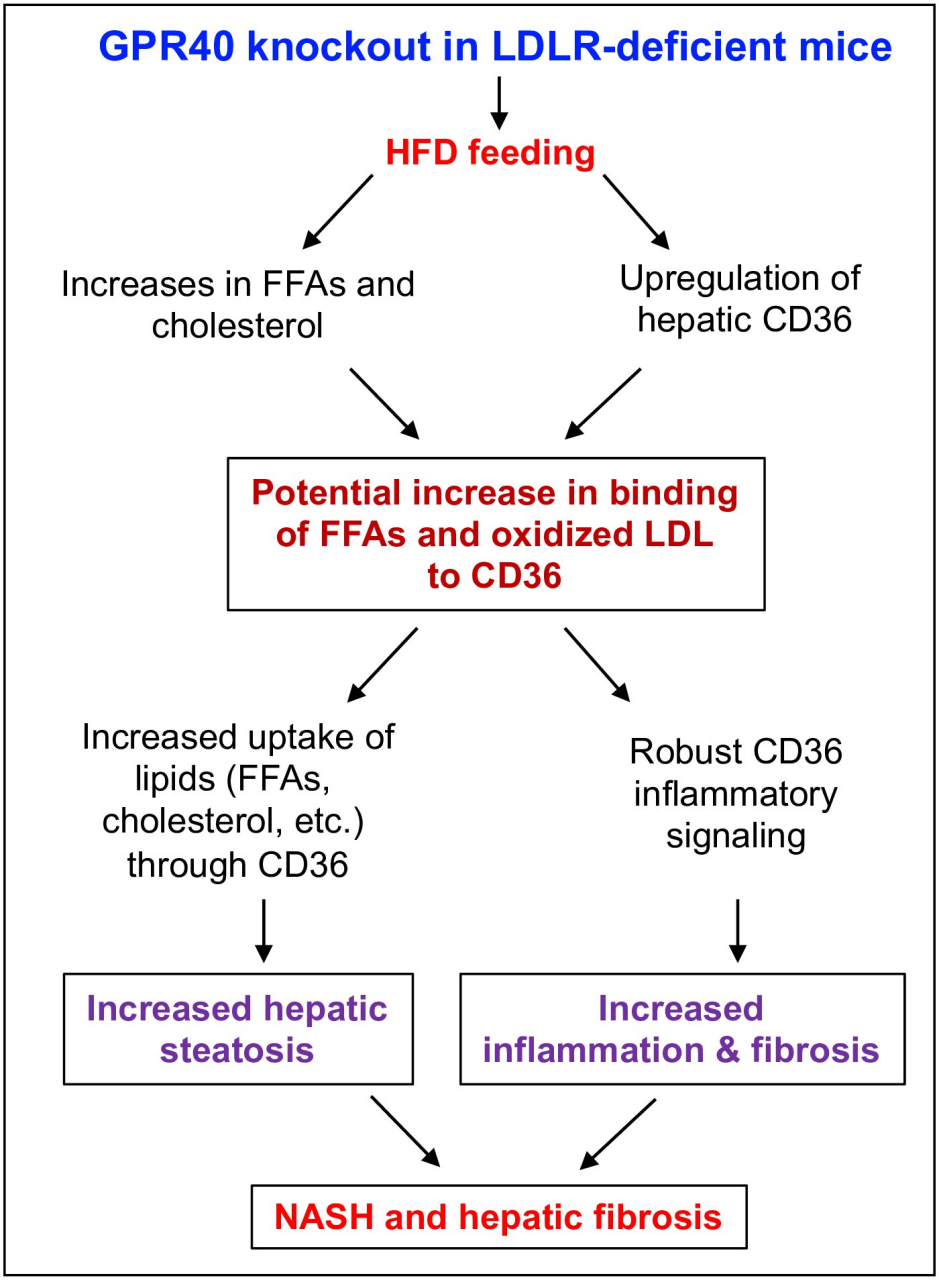
The proposed mechanism by which GPR40 KO led to increased hepatic steatosis, inflammation and fibrosis in LDLR-deficient mice. Since GPR40 KO augmented both plasma FFAs and hepatic CD36 expression, the CD36-dependent uptake of FFA in liver is likely increased. Additionally, since most cholesterol is associated with LDL and LDL oxidation is boosted by metabolic syndrome, oxidized LDL is increased in HFD-fed LDLR KO mice. As oxidized LDL is a ligand for CD36, oxidized LDL is also likely to be taken up by CD36 to increase lipids in liver. Furthermore, the engagement of FFAs and oxidized LDL with CD36 activates CD36 proinflammatory signaling, leading to hepatic steatosis, inflammation and fibrosis.

The findings that GPR40 KO led to increased levels of FFA and cholesterol and hepatic CD36 upregulation in LDLR -deficient mice reveal a plausible mechanism by which GPR40 KO increases HFD-induced hepatic steatosis, inflammation and fibrosis. As illustrated in Fig 10, since GPR40 KO augmented both plasma FFAs and hepatic CD36 expression, the binding of FFAs to CD36 is likely to be enhanced, leading to an increased uptake of FFAs. Additionally, since most cholesterol is associated with LDL and the oxidation of LDL is promoted by MetS [58], it is likely that the high level of cholesterol in HFD-fed LDLR-deficient mice would increase production of oxidized LDL [59]. As oxidized LDL is a ligand for CD36 [60], it would bind to CD36 to increase lipid uptake in liver, contributing to steatosis. Moreover, the engagement of FFAs and oxidized LDL to CD36 is likely to trigger CD36-mediated proinflammatory signaling, resulting in hepatic inflammation and fibrosis.

The finding that GPR40 KO increases the plasma levels of cholesterol and FFAs suggests that GPR40 signaling controls the production of cholesterol and FFA in liver. This finding is in line with the previous reports that GPR40 signaling is involved in the modulation of cholesterol and FFA production [61, 62]. Li et al. have shown that GPR40 agonist inhibits the expression of hepatic lipogenic genes by stimulating AMP-activated protein kinase (AMPK) signaling in hepatocytes [61]. On et al. have also shown that docosahexaenoic acid (DHA) inhibits hepatic expression of lipogenic genes such as SREBP-1, a key lipogenic transcription factor, through GPR40 [62]. In our current study, we have also demonstrated that GPR40 KO is associated with increased hepatic expression of SREBP-1c and FAS, which play a crucial role in the production of cholesterol and FFAs [63, 64]. Our study further showed that GPR40 KO is also associates with decreased hepatic expression of CPT1a and ACOX1, which are known to be involved in fatty acid oxidation and turnover [65]. It has been shown that downregulation of genes involved in fatty acid oxidation and turnover is associated with hepatic steatosis [66].

A surprising finding from our previous study using C57BL/6 mice as animal model is that GPR40 KO led to hepatic steatosis, inflammation and fibrosis in LFD-fed mice [13]. Interestingly, we have made the similar observation in LFD-fed LDLR-deficient mice in the current study. To explore the underlying mechanisms, we found that similar to LFD-fed C57BL/6 mice, GPR40 KO also upregulated hepatic CD36 expression in LFD-fed LDLR-deficient mice. These findings strongly suggest that although LFD is not associated with hyperlipidemia, the upregulation of CD36 by GPR40 KO increases lipid uptake and hepatic inflammation, contributing to hepatic steatosis, inflammation and fibrosis. Consistently, we showed that GPR40 KO in LFD-fed LDLR-deficient mice increased hepatic expression of aSMA, TGFβ1, IL-6 and TNFα, which are key molecules involved in hepatic fibrosis and inflammation [67, 68].

The findings from the present study indicate that GPR40 and CD36 are fundamentally different in the regulation of inflammation that is crucial in the progression of NAFLD. In our *in vitro* studies using BMMs isolated from wild-type or GPR40 KO mice, we demonstrated that loss of GPR40 led to the upregulation of IL-6 by PA through a CD36-dependent mechanism. We also showed that inhibition of CD36 with CD36 inhibitor SSO nearly abolished IL-6 expression and secretion stimulated by LPS or LPS plus PA, but inhibition of GPR40 had no effect. Furthermore, we showed that GPR40 agonism inhibited expression of proinflammatory genes such as IL-6, TNFα and MCP-1 in macrophages. Clearly, all these studies revealed anti-inflammatory properties of GPR40 in macrophages, which is consistent with the previous studies reporting the anti-inflammatory effects of GPR40 in microglia, pancreatic beta cells and hypothalamus [9, 69, 70]. In addition, the anti-inflammatory effect of GPR40 is also indicated by the fact that while both GPR40 and CD36 are the receptors for long-chain FFA, GPR40 is also a receptor for medium-chain FFA [9]. It has been shown that medium-chain FFAs inhibit inflammatory responses in microglia through GPR40-dependent pathway [9].

It has been reported that PA is also a Toll-like receptor (TLR) 4 agonist [71]. Since we have shown previously that LPS potently induced proinflammatory cytokine expression from BMMs [39], it suggests the presence of TLR4 on BMMs. It is expected therefore that PA is capable of stimulating proinflammatory cytokine expression via TLR4. However, we found in this study that PA failed to stimulate IL-6 secretion from BMMs prepared from wild-type mice. The above observation is consistent with our previous report that PA did not stimulate IL-6 mRNA expression in BMMs [39] and murine macrophages [41]. Interestingly, we found that although PA did not stimulate IL-6 expression, it upregulated other proinflammatory molecules such as IL-1α, CXCL10, and CD86 [39]. Nevertheless, it is noteworthy that the upregulation of proinflammatory molecules by PA was much weaker than that by LPS. Due to the weak stimulation of proinflammatory molecule expression by PA, the reports on the activation of TLR4 by PA remains controversial. On the one hand, it was reported that free fatty acids including PA did not activate TLR2 and TLR4 in macrophages [72]. On the other hand, it was reported that SFAs including PA activated TLR-mediated proinflammatory signaling pathways [73]. Therefore, further investigations to define the role of PA in TLR4 activation are warranted.

In conclusion, the current study showed for the first time that GPR40 KO in LDLR-deficient mice worsened HFD-induced hepatic steatosis, inflammation and fibrosis by potentially increasing FFAs and cholesterol and upregulating hepatic CD36 expression. This study also showed that GPR40 had anti-inflammatory properties in macrophages, suggesting that GPR40 is a potential therapeutic target for hyperlipidemia-associated NASH.

## Supporting information

S1 Checklist (PDF)
